# Current Trends and Promising Electrode Materials in Micro-Supercapacitor Printing

**DOI:** 10.3390/ma16186133

**Published:** 2023-09-09

**Authors:** Tatiana L. Simonenko, Nikolay P. Simonenko, Philipp Yu. Gorobtsov, Elizaveta P. Simonenko, Nikolay T. Kuznetsov

**Affiliations:** Kurnakov Institute of General and Inorganic Chemistry, Russian Academy of Sciences, 119991 Moscow, Russia; egorova.offver@gmail.com (T.L.S.); phigoros@gmail.com (P.Y.G.); ep_simonenko@mail.ru (E.P.S.); ntkuz@igic.ras.ru (N.T.K.)

**Keywords:** carbon-based materials, transition metal oxides, polymers, transition metal hydroxides, metal sulphides, MXenes, electrodes, printing technologies, micro-supercapacitors

## Abstract

The development of scientific and technological foundations for the creation of high-performance energy storage devices is becoming increasingly important due to the rapid development of microelectronics, including flexible and wearable microelectronics. Supercapacitors are indispensable devices for the power supply of systems requiring high power, high charging-discharging rates, cyclic stability, and long service life and a wide range of operating temperatures (from −40 to 70 °C). The use of printing technologies gives an opportunity to move the production of such devices to a new level due to the possibility of the automated formation of micro-supercapacitors (including flexible, stretchable, wearable) with the required type of geometric implementation, to reduce time and labour costs for their creation, and to expand the prospects of their commercialization and widespread use. Within the framework of this review, we have focused on the consideration of the key commonly used supercapacitor electrode materials and highlighted examples of their successful printing in the process of assembling miniature energy storage devices.

## 1. Introduction

The creation of efficient and environmentally friendly energy storage systems is currently one of the most important tasks of material science in the field of global energy, becoming more and more important due to the annually growing level of energy consumption and anthropogenic impact on the environment. In this context, the special attention of scientists and engineers is drawn to supercapacitors, also called ionistors or electrochemical capacitors, which occupy an intermediate position between traditional (electrolytic) capacitors and lithium-ion batteries, demonstrating higher values of energy density in comparison with the first type of devices and higher values of specific power in relation to the second type of devices. In addition, other advantages of supercapacitors over lithium-ion batteries include higher Coulombic efficiency (85–98% vs. 75–90% for lithium-ion batteries) and charge–discharge rate (from a few seconds to a few minutes vs. 0.3–3 h in rechargeable batteries), longer service life (more than 500,000 operating cycles versus about 1000 cycles in rechargeable batteries), and a wider range of operating temperatures (from −50 to 50 °C while Li-ion batteries lose about 50% of their capacity already at −20 °C) [1,2].

Based on the type of charge storage mechanism, supercapacitors can be divided into double-layer and pseudo-capacitors. Devices of the first type store charge in the double electric layer arising on the electrode–electrolyte boundary by means of reversible physical processes of ion adsorption in this area, whereas in pseudocapacitors, the process of charge accumulation occurs both due to the work of the double electric layer and due to the phenomenon of pseudocapacitance, namely the course of reversible redox chemical reactions in the near-surface (at a depth of a few nanometers) layers of electrodes. The described effect results in higher capacitive characteristics as well as power density values of pseudocapacitors compared to double-layer supercapacitors [3,4].

One of the main application areas of supercapacitors is microelectronics, which, as we know, is currently striving for a paradigm shift as part of the transition from rigid to flexible printed circuit boards, functional and structural elements, and their miniaturization. The development of flexible, compact, portable microelectronics devices, including wearable devices, requires the creation of flexible electrical energy storage systems, including those based on supercapacitors, while maintaining their mechanical strength and high values of performance characteristics (specific capacity and power, charge-discharge rate, cyclic stability, number of operation cycles) [5,6]. To date, the search for different variants of geometric design of supercapacitors is underway in order to achieve the flexibility and compactness of their assembly. One of the most common types of configurations of these devices are the so-called sandwich structures, in which the electrolyte layer is located between two layers of electrodes. This type of assembly is widespread in the creation of volume-type supercapacitors due to the simplicity of design, low cost of its manufacture, and wide scaling possibilities. However, with this multilayer configuration, the electric current flows perpendicular to the entire assembly, resulting in a significant resistance between the different layers as well as an increase in the ion diffusion path length, all of which leads to lower specific capacitance and power density values of the final device [7]. In addition, when such a supercapacitor is bent, its functional layers may be displaced relative to each other, which is critical for flexible electronic devices [8]. An improved alternative to the described sandwich structures is the creation of planar structures with interdigital electrodes located in the same plane, the surface of which and the distance between which is covered by a layer of gel-polymer electrolyte. This geometrical implementation allows for a significant reduction in the thickness, an increase in the flexibility of the supercapacitor cell, and a reduction in its size while eliminating the mutual displacement of its layers. The presence of interdigital electrodes, standing apart from each other in one plane at a small distance, allows us to exclude the possibility of a short circuit between them, reduce the electrical resistance between the layers and reduce the length of the path of diffusion of ions, contributing to more active processes of interaction at the electrode–electrolyte interphase boundaries, which significantly improves the electrochemical characteristics of the final supercapacitor [9,10].

The formation of flexible planar micro-supercapacitors (MSCs) with complex geometries requires advanced high-tech methods and approaches. Thus, photolithography, vacuum sputtering, and laser engraving methods are used to solve this problem [11,12,13,14,15,16], but the complexity, high cost, and limited scalability of these approaches impede their wide application. Recently, in the formation of various electronics and alternative energy devices, including supercapacitors, the trend towards the use of additive technologies (3D printing [17], ink-jet printing [18], aerosol printing [19], pen plotter printing [20,21], microplotter printing [22], microextrusion printing [23], roll-to-roll printing [24], transfer printing [25], and screen printing [26]) is observed, which allow for the automated formation of structures of different (including complex) geometries with a high degree of reproducibility, dosing accuracy, and targeted material application as well as the provision of an opportunity to effectively scale the process while maintaining the microstructural and functional characteristics of manufactured devices.

The choice of substrate material is an important task in the formation of flexible planar type supercapacitors using printed technologies. In the context of these methods, parameters, such as the flexibility, thickness, roughness of the substrate as well as its density and thermal resistance, must be considered. The substrate wettability is an important parameter because it has a significant influence on the microstructure of the formed coatings during printing [27]. For example, printing with water-based inks on the surface of hydrophobic substrates will be difficult due to the inhomogeneity of ink distribution on their surface [28]. The four most common types of substrates in the fabrication of flexible supercapacitors are: those based on paper, textile, metal foil, and polymer. Paper substrates should be highlighted as one of the most promising types of substrates from the point of view of printed technologies for the formation of supercapacitors since they have good wettability with a wide range of solvents as well as the necessary flexibility and commercial availability, and importantly, they are more environmentally friendly and biodegradable than polymer substrates [18]. Current research shows that this type of substrate allows for the formation of assemblies of high-efficiency planar-type supercapacitors [29].

The set of functional elements of planar type supercapacitors with interdigital electrodes is similar to that for sandwich-type supercapacitors: current collectors, working electrodes, and the electrolyte layer. As a rule, materials with high electrical conductivity are used as current collectors: metals (stainless steel, silver, gold, etc.), conductive polymers (e.g., poly-3,4-ethylenedioxythiophene:polystyrene sulfonate—PEDOT:PSS), and various carbon materials (graphite, carbon nanotubes, reduced graphene oxide, etc.) [30,31,32]. It should be noted that carbon materials of current collectors, in addition to high electrical conductivity, environmental friendliness, and availability, are also characterized by a high specific surface area, which in turn provides better contact and charge transfer at the “current collector—working electrode” interphase boundaries [7]. An important component of supercapacitors is the electrolyte, the selection of which is based on the composition of the functional components of the device. More detailed discussion of the types of electrolytes used for micro-supercapacitor assembly will be given in Section 2.3.

The choice of MSCs electrode material is extremely important in terms of the desired electrochemical performance of the final device (specific capacitance value, cyclic stability, charge-discharge rate, power density and energy density, and cyclic stability and lifetime). According to the SciFinder database, from 1952 to 2022 (only 4658 papers have been indexed so far in the first eight months of 2023), 74,266 publications of various types (mostly research and review journal articles, patents, conference abstracts, and books) containing the keyword “supercapacitor” have been indexed. With each year the number of publications on this subject is steadily increasing. However, when refining the query with the keyword “printing” the number of works sharply decreases (down to 935), which indicates the high novelty of developments in the field of printed supercapacitors and their miniaturization. The most promising and sought-after materials for printing electrodes of these devices, according to the analysis of the results of the search, are such groups of materials as carbon and its derivatives, metal oxides and hydroxides, polymers, metal sulfides, composites based on them, and a new and little-studied class of 2D-materials—MXenes. Thus, this review focuses on the prospects for the application of the above groups of materials in the context of printing electrodes for modern flexible/wearable miniature supercapacitors.

## 2. The Most Common Electrode Components of Printed Micro-Supercapacitors

### 2.1. Carbon Materials

Carbon and its allotropic modifications are currently one of the most important elements in the development of advanced microelectronics [33,34], biosensors [35,36,37], and alternative energy applications [38,39]. In the development of energy storage devices, carbon materials, due to their high specific surface area, high electrical conductivity, and chemical stability, are more and more actively used for the development of electrodes that store charge via electrical double layer mechanism. Among the most widely explored varieties of carbon materials in terms of printing electrode structures for micro-supercapacitors are onion-like carbons, carbon nanotubes, graphene, carbon aerogels, and activated carbon [40].

In the study by Zeiger et al. [41], it is proposed to apply the concept of onion-like carbons (OLC) only to those carbon particles that have at least four shells (by analogy with multi-walled carbon nanotubes) are characterized by a size less than 100 nm, have a spherical or polyhedral shape and partially defective structure (amorphous domains or islands of sp^3^-hybridized carbon are present). This category of materials includes both hollow onion-like carbons and those whose core consists of metal clusters or fragments of nanodiamonds left inside the carbon onions as a result of their incomplete transformation. When referring to onion-like carbons less than 10 nm in size, the term “carbon nano-onions” (CNO) may be used [41,42]. There are several approaches to the synthesis of these materials, but the most effective one is considered to be the thermal treatment of detonation nanodiamonds, which allows us to achieve a high practical yield of particles smaller than 10 nm with a high degree of carbon ordering, the value of the specific surface area for which can reach 600 m^2^/g. Basic electrodes based on CNO and OLC, as a rule, demonstrate rather low values of specific capacitance (less than 100 F/g) [43] and specific energy, but are characterized by high conductivity (up to 4 S/cm) and charge/discharge rate. In connection with this, various attempts are made to physically and chemically activate these materials as well as to decorate them with redox-active substances in order to further optimize their electrochemical characteristics [43]. In addition, CNO and OLC can act as conductive additives in hybrid electrodes [44]. Currently, there are not many works on ink production and investigation of the possibility of CNO and OLC application in the printing of electrode structures. In particular, in the work of Cumba et al. [45], CNO and graphite-based inks were fabricated with subsequent screen printing of working electrodes on the surface of PET substrates. The coatings thus formed showed an ohmic resistivity value of 800 ± 24 Ω/cm and a heterogeneous electron transfer rate constant of 1.3 ± 0.7 × 10^−3^ cm·s^−1^. Pinto et al. [46] considered the prospects of inkjet printing of planar electrodes using inks that are dispersions of CNO in ethanol (solid phase concentration 6 mg/mL; viscosity ∼1.2 mPa∙s). The authors were able to obtain inks compatible with a commercial inkjet printer with a piezoelectric printhead and to print conductive tracks with a thickness of about 180 nm, a width of about 256 μm (cross section of about 36 μm) in 10 printing cycles (220 μm resolution), characterized by a resistivity of 600 Ω·m and a negative temperature coefficient of resistance of about −4.35 × 10^−2^ °C^−1^.

Carbon nanotubes (CNT) due to their high electrical conductivity (5 × 10^5^ S/m [47]), mechanical strength, and relatively high specific surface area (the theoretical value of this parameter can reach 1315 m^2^/g for single-walled CNTs [48]) are also of considerable interest in the context of electrode materials for supercapacitors. It is reported [49] that the specific capacitance of electrodes based on unmodified single- and multi-walled CNT does not exceed 45 and 80 F/g, respectively. In order to achieve more impressive functional characteristics (specific capacitance, energy density, and charge/discharge rate), strategies such as functionalization of CNT with various conductive materials (graphene, metal nanoparticles, transition metal oxides, MXenes) as well as their deposition on thin metallic films are employed [50]. Fibers [51] and fabrics made of intertwined nanotubes [52] are actively used in the creation of flexible and wearable supercapacitors. Approaches, such as 3D printing [53], ink-jet printing [54], screen printing [55], and direct ink writing (DIW) [56], have been used to print CNT-based supercapacitors. Thus, S. Ryan et al. [47] report the 3D printing of an asymmetric supercapacitor consisting of a SWCNT/SnO composite positive electrode and a negative electrode based on Ti_3_C_2_T_x_ multilayer MXene. SWCNT/SnO ink (SWCNT:SnO = 9:1) was made by dispersing 0.2% active material and 0.3% carboxymethylcellulose in water. The nozzle diameter for printing was 0.26 mm, and the speed was 200 mm/min; the electrode structures were deposited on the surface of the glass substrate. The authors also report that the use of SnO additives in the ink composition allowed it to inhibit the oxidation of nanotubes and widen the operating potential window (up to 1.4 V in H_2_SO_4_ and K_2_SO_4_) while maintaining 95% capacity after 7500 cycles at a current density of 10 A/g. In [56], the possibilities of direct ink writing for forming functional layers of a flexible supercapacitor on the surface of a polyimide substrate as well as the usage of a sealing layer based on silicon rubber for creating a fully packaged device were demonstrated. An ink based on SWCNT (32 mg) and sodium lauryl sulfate (120 mg) in water (40 mL) was used to print the active electrode material, and a viscous ink based on polyvinyl alcohol (6 g) and lithium chloride (12 g) in water was used to print the electrolyte. The nozzle diameter for forming electrode structures was 0.2 mm, and the printing speed was 200 mm/s. This printed supercapacitor was characterized by rather high specific capacitance (15.34 F/cm^3^ at 1.32 A/cm^3^), energy density 1.18 mWh/cm^3^ at power density 11.8 W/cm^3^, and high cyclic stability (97.7% of capacitance retention after 1000 cycles). The authors of [55] consider screen printing an efficient and inexpensive approach to create flexible all-in-one supercapacitors based on multi-walled carbon nanotubes printed on the surface of cotton fabric. The formed devices were characterized by a specific capacitance of 4.16 mF/cm^2^, high cyclic stability with 97.4% capacitance retention after 1000 bending/flexing cycles, and high bending strain rates of about 20% s^−1^.

**Figure 1:**
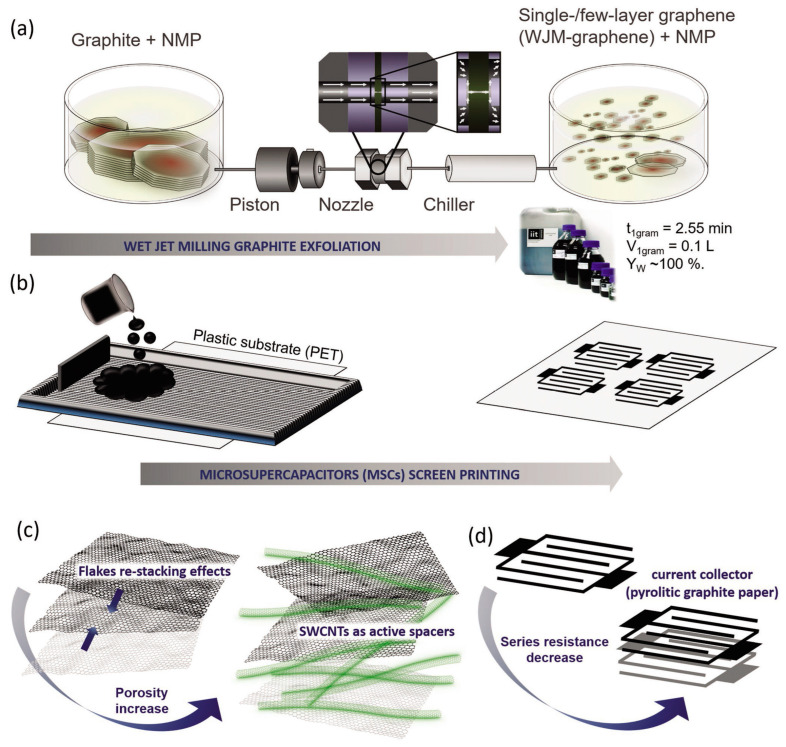
**(a**) Schematic illustration of the production of single-/few-layer graphene by WJM exfoliation of graphite (WJM-graphene). (**b**) Screen printing of MSCs onto plastic substrate (PET). (**c**) Addition of SWCNTs as active spacers for avoiding the re-stacking of the flakes. (**d**) Use of pyrolytic graphite (PG) paper in order to decrease the current collector resistance of MSCs for high-power density requirements. Reproduced from Ref. [61]. Copyright 2019, WILEY-VCH Verlag GmbH & Co. KGaA, Weinheim, Germany.

Graphene and materials based on it (graphene oxide and its reduced form) do not lose their popularity in the field of electrode formation of electrochemical energy storage devices. It is known that graphene is characterized by outstanding values of specific surface area (up to 2630 m^2^/g) and charge carrier mobility (carrier mobility of up to 2 × 10^5^ cm^2^·V^−1^·cm^−2^), as well as a fairly high specific capacitance compared to other carbon materials (about 550 F/g). Currently known varieties of graphene microstructures include wrinkled graphene, porous graphene, graphene nanomeshes, honeycomb-like graphene, graphene hydrogels, and 3D porous graphene [57]. Additionally, in order to improve its electrochemical characteristics, the approach of combining it with other materials to create hybrid electrodes (CNT, conducting polymers, transition metal oxides, and hydroxides) is being actively used [58,59]. According to a review by Mensing et al. [60], the most widely used approaches for printing graphene-based nanostructures for supercapacitors are inkjet printing, screen printing, and 3D printing. The authors also specify that it is preferable to obtain graphene for the manufacture of functional inks using top-down methods, which are based on exfoliation or chemical modification of bulk carbon materials (e.g., graphite), which allows to obtain dispersions of single or few layers of graphene in a liquid medium. The resulting dispersions can be used directly as inks or further modified to optimize viscosity, surface tension, and stability. For example, Bellani et al. [61] demonstrated a productive approach to graphite exfoliation in N-methyl-2-pyrrolidone using the wet-jet milling technique, which allowed for the formation of large volumes (at the liter level) of concentrated (grams per liter) dispersions based on single and low-layer graphene sheets (Figure 1). Based on graphene thus obtained, functional inks were prepared in a mixture of water and ethanol (70:30) with the addition of 1 wt.% terpineol and 25 wt.% SWCNT for subsequent screen printing of the micro-supercapacitor on the surface of the polymer substrate. The obtained devices exhibited high volumetric capacitance (0.490 F/cm^3^) as well as power density values above 20 mW/cm^2^ with an energy density of 0.064 μW/cm^2^. In addition, the printed micro-supercapacitor was characterized by high cyclic stability with 97% capacity retention after 10,000 charge/discharge cycles as well as resistance to bending and folding [61].

Widely commercially available materials for the manufacture of supercapacitors on an industrial scale are activated carbon, obtained by physical or chemical activation of carbon-containing materials, such as bamboo, willow peat, wood, plant biomass (fibers, husks, shells), fossil coal, and petroleum pitch. Physical activation involves carbonization in an inert environment (at <1000 K) followed by heat treatment (>1150 K) of the resulting coal in an environment of activating gaseous agents (CO_2_, water vapor, air, or a combination thereof). Chemical activation is a one-stage process of carbonization of initial carbon-containing materials (700–1200 K) in the presence of activating agents (hydroxides of alkali and alkaline-earth metals, acids, and some salts) [62]. Bora et al. [63] consider methods of formation of activated coals on the basis of varieties of fossil coal. It is noted that selection of conditions of the chemical activation of coal allows to form 3D carbon materials with hierarchically organized pore structure (including carbon aerogels), to achieve high values of specific surface area (about 3000 m^2^/g), significant values of specific capacitance (up to 400 F/g), and cyclic stability of formed final supercapacitors. When printing planar micro-supercapacitors based on such materials, 3D printing (in particular Direct Ink writing) and screen-printing work are most common. Thus, in [64], a successful DIW formation of a micro-supercapacitor was shown which demonstrated a high specific capacitance (1892.90 mF/cm^2^ at 0.3 mA/cm^2^) as well as a bulk energy density of 9.20 mW·h/cm^3^ at a power density of 6.89 mW/cm^3^. Jost et al. [26] presented a flexible supercapacitor formed by screen printing activated carbon on knitted carbon fiber fabric, which reached capacitance values of 510 mF/cm^2^ at 10 mV/s.

### 2.2. Transition Metal Oxides

Nowadays, transition metal oxides in the context of electrode components of electricity storage devices are represented very widely. This class of materials is characterized by high electrochemical activity: its representatives can participate in reversible redox reactions (Faraday processes), providing additional (besides the work of the double electric layer) energy storage (pseudo-capacitive effect) due to the presence of metals with several oxidation degrees in their composition [65]. Ruthenium oxide stands out among transition metal oxides due to its high capacitive characteristics (the value of theoretical specific capacitance is 1400–2000 F/g), but the known problems of this material, such as is its high cost and high tendency to agglomeration in the process of long-term operation, significantly reduce its prospects for widespread use. In this connection, various options for creating RuO_2_-based composites are currently being considered in order to “dilute” it with cheaper components while maintaining high electrochemical characteristics. In particular, the characteristics of ruthenium oxide composites with carbon materials, polymers, and other transition metals are being investigated [66]. Another approach to reducing the cost of devices based on ruthenium oxide is the reduction of its material intensity during planarization and miniaturization of electrodes based on it. Thus, Huang et al. [67] proposed a convenient approach to fabricating MSCs consisting of RuO·xH_2_O electrode and platinum current collectors deposited on the surface of a polymer substrate. A standard consumer inkjet printer was used to print the sacrificed patterns required to delineate the applied functional layers. It was shown that the final device exhibited outstanding electrochemical performance, with a bulk power density of 73,460 mW/cm^3^ and an energy density of 24.9 mW·h/cm^3^.

Other materials that attract the increased attention of researchers in the context of the formation of electrode nanostructures are oxides of manganese [68], iron [69], nickel [70], and cobalt [71]. Thus, manganese is capable of forming a wide range of MnO_x_ oxides (MnO, Mn_2_O_3_, Mn_3_O_4_, Mn_5_O_8_, MnO_2_) with different crystal structures, which are of interest not only in the field of electrochemical energy, but also in catalysis as well as optics and sensorics [20]. Manganese oxides both in individual form (e.g., δ-MnO_2_ [72], MnO_2_ [73]) and in the form of composites with carbon [74,75] and other [76] materials are actively used as component in functional inks for printing MSCs electrodes. Thus, in a work by Wang et al. [72], interdigital electrode structures were deposited using ink based on δ-MnO_2_ nanosheets (lateral size 89 nm, thickness—about 1 nm) in a mixture (1:10) of propylene glycol and water by inkjet printing on the surface of polyimide film (thickness—120 μm). The symmetric supercapacitor obtained on their basis showed high volume capacity (2.4 F/cm^3^) and cyclic stability with 88% capacity retention after 3600 operating cycles. Significant improvements in the performance of printed manganese oxide-based electrodes can be achieved when it is combined with other materials. In particular, Mokhtarnejad et al. [75] showed the high efficiency of supercapacitors formed entirely by 3D printing, containing MnO_x_/Mn_3_O_4_–rGO composite as an active electrode material. As a result, the authors were able to achieve an increased (325 F/g) specific capacitance of the device compared to supercapacitors based on commercial Mn_3_O_4_–rGO (189 F/g).

A considerable number of works have been devoted to the study of the possibilities of using iron oxides (FeO_x_) as electrode materials for energy storage devices [77,78]. These materials have the advantages of high redox activity, high theoretical capacity, commercial availability, and low environmental impact. Despite their high energy and power density, they have several disadvantages, specifically low conductivity and a short lifetime due to low structural stability during cyclic volume changes occurring upon charge and discharge of the supercapacitor [69]. In a number of works [79,80], in order to eliminate the indicated problems of iron oxides, a combination with carbon materials is proposed, as was in the case of manganese oxides. In particular, Zhang et al. [80] reported the formation of an effective supercapacitor with the screen printing of rGO/Fe_2_O_3_ electrode structures (the printing ink was a dispersion of the corresponding particles in the ethylcellulose-ethanol system). The resulting device exhibited excellent performance during testing, with capacitance values in the range of 27.12–119.37 mF/cm^2^ at 0.1 mA/cm^2^ and energy density values of 2.4–10.6 µW·h/cm^2^ at a power density of 0.04 mW/cm^2^ as well as exceptional flexibility with no loss of capacitance after 200 bending cycles.

Cobalt oxides as well as nickel oxide do not lose their high relevance as components of supercapacitors and batteries due to high values of theoretical specific capacitance (Co_3_O_4_: 3560 F/g [81]; NiO: 2584 F/g [82]), chemical and thermal stability, low toxicity, and their commercial availability [83,84,85]. Researchers combine various synthetic approaches and printing techniques to form electrode structures of complex geometry with a diversified, often hierarchically organized, microstructure, which makes a significant positive contribution to the desired electrochemical characteristics of the resulting planar nanostructures [86,87]. Among others, Dutta et al. [88] investigated the effect of calcination temperature in the range of 300–800 °C on nickel hydroxide and the functional characteristics of micro-supercapacitors fabricated by screen printing of the corresponding NiO inks (diacetone alcohol (91%)–cellulose acetate propionate (9%) system was used as a dispersion medium) on a polymer substrate. It was found that the electrode structures formed with the use of ink based on nickel oxide obtained at 400 °C (NiO-400) and characterized by a continuous yet porous structure showed the highest specific capacitance—19.54 mF/cm^2^. The asymmetric supercapacitors based on NiO-400 cathode and graphene anode exhibited a high specific capacitance of 26.42 mF/cm^2^ over a wide potential range of 1.5 V (with a Coulomb efficiency of 97.3%) and were characterized by excellent flexibility with 96.4% capacitance retention (180°) after 1000 cycles.

**Figure 2:**
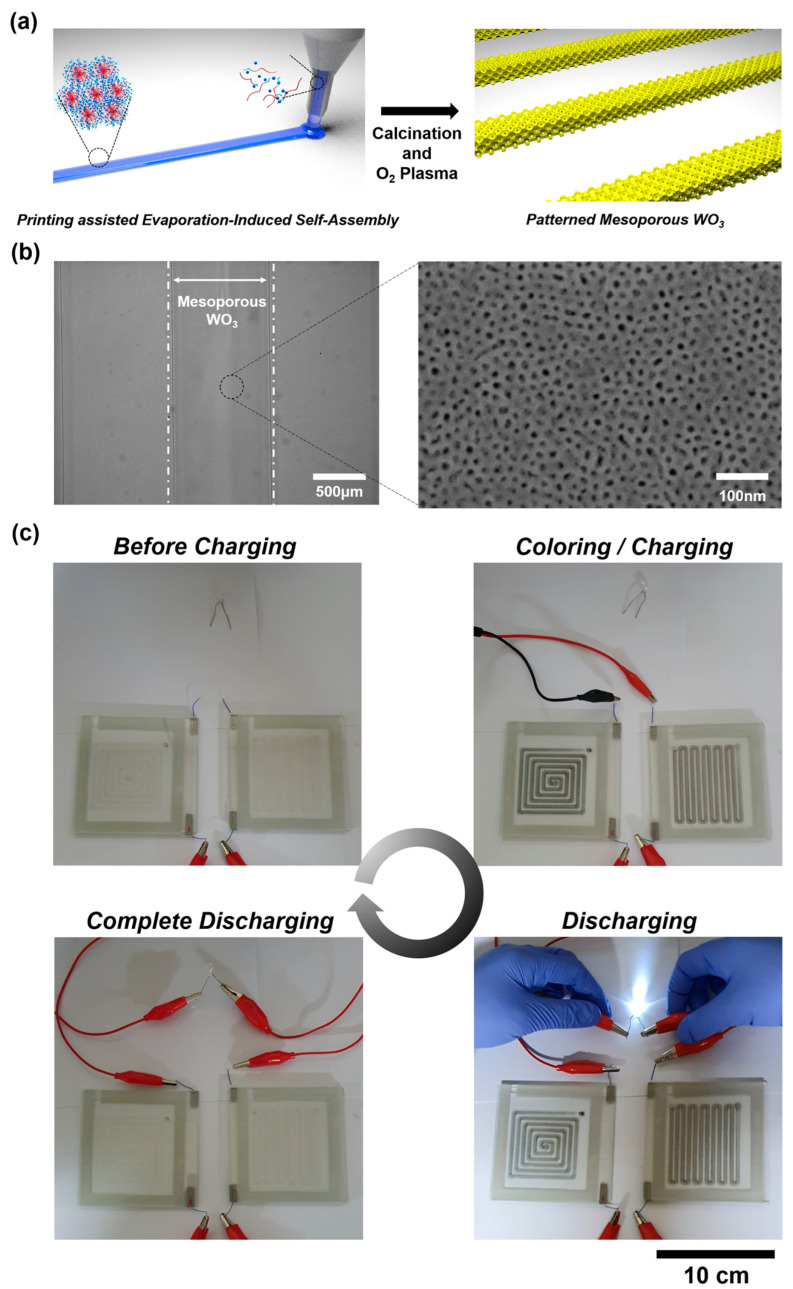
Application of PEISA for the fabrication of functional ECSDs. (**a**) Schematic illustration of PEISA. (**b**) OM (left) and SEM (right) images of mesoporous WO_3_ fabricated by PEISA. (**c**) Photographs of the ECSD during the reversible charging (coloration of the pattern) and discharging (LED light on and bleaching) test. For this application, two ECSDs were connected in series. Reproduced from Ref. [91]. Copyright 2019, Kim et al.

Recently, vanadium (most commonly V_2_O_5_) and tungsten (WO_3_) oxides have also attracted attention. V_2_O_5_ is characterized by a layered structure into which electrolyte ions can reversibly intercalate (which gives a total capacity of 440 mA·h/g) and is considered an electrode of pseudocapacitors with a wide potential window of 1.5–3.8 V [89]. WO_3_ in its turn possesses high electronic conductivity, high packing density, and high energy density [90]. Both of these oxides have electrochromic properties, which allows for their application in special electrochromic supercapacitors. Kim et al. examined the possibility of creating such a device based on amorphous mesoporous tungsten trioxide via printing-assisted evaporation-induced self-assembly (Figure 2) [91]. The obtained electrochromic supercapacitor showed high values of specific capacitance of 2.57 mF/cm^2^ at a current density of 1.0 mA/cm^2^ and an optical modulation of 76% at 700 nm along with high coloration/bleaching rates (0.8 s for coloration and 0.4 s for bleaching). Yilmaz et al. [92] developed solid-state supercapacitors based on V_2_O_5_/CNT composite electrodes with high energy density and excellent cyclic stability. The electrodes were deposited using a screen printing method on the surface of In_2_O_3_-SnO_2_ film glasses, and inks with different mass ratios of V_2_O_5_/CNT (0.1:1, 0.5:1, 1:1, 1:1, 2:1, and 5:1) were used. The final electrode structures were characterized by a hierarchically organized pore structure promoting facilitated diffusion of electrolyte ions to the active centers on the surface and improved electron transport. The resulting miniaturized supercapacitor with LiCl/polyvinyl alcohol electrolyte provided high energy density (1.47 mWh·cm^3^) and power density (0.27 W·cm^3^). It was also observed to retain 91% of capacity after 5000 cycles of operation at a charge–discharge current of 5 A/g.

A further step towards the development of supercapacitor electrodes based on transition metal oxides was the use of oxides of complex composition, combining the advantages of individual oxides. Materials with spinel-type structure based on cobaltites, ferrites, and metal manganites are being especially actively investigated (e.g., NiCo_2_O_4_, NiFe_2_O_4_, ZnCo_2_O_4_, MgCo_2_O_4_, CuCo_2_O_4_, ZnMn_2_O_4_, and CoMn_2_O_4_) [93,94,95,96]. Other promising candidates for the role of electrode material are considered to be molybdates with the general formula AMoO_4_, combining high values of electrical conductivity (about 1.13 × 10^−4^ S/cm at 300 K) as well as thermal and mechanical resistance. The combination of several different materials of this family (e.g., NiMoO_4_ and CoMoO_4_) in the electrode composition allows it to achieve an additional increase in capacitance values (about 1200 F/g) [97]. There are also successes in the field of printing supercapacitors based on such materials. Shinde et al. [98] used a chemical coprecipitation approach to form flower-like NiCo_2_O_4_/NiCo_2_S_4_ nanopowders and subsequently produce inks based on them (polyvinyl alcohol was also used as a component). Next, electrode films of appropriate composition were formed on the Ni mesh surface using screen printing. Electrochemical tests showed that the final device exhibited a specific capacitance of 130 F/g at 5 mV/s [98]. 

**Figure 3:**
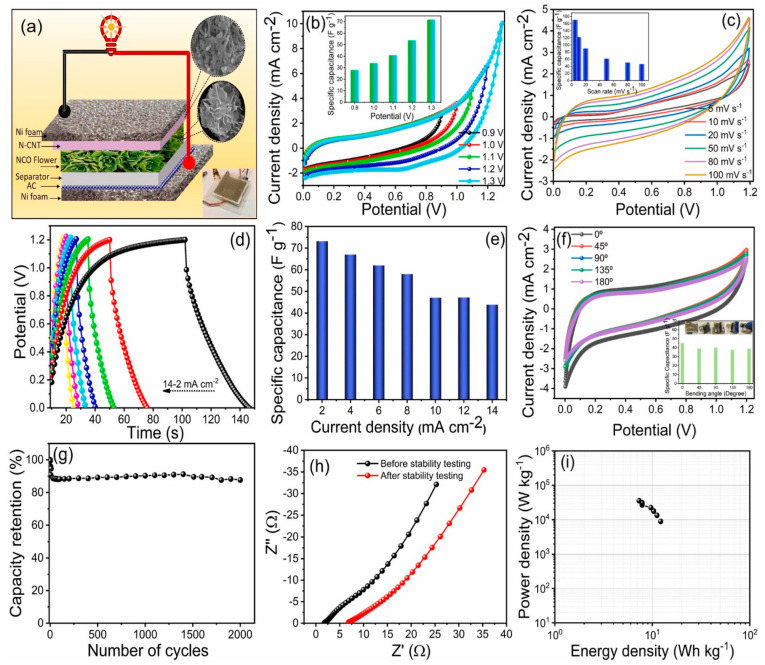
(**a**) Schematic assembly of SSHSc device, inset shows the real images of the device; (**b**) potential window optimization from CV measurements and inset shows the specific capacitance with potential windows; (**c**) CV curves with different scan rates from 5 to 100 mV s^−1^, and inset shows the specific capacitance with respect to scan rates; (**d**) GCD curves with different current densities; (**e**) specific capacitance with respect with different current densities; (**f**) bending angle study and inset shows the specific capacitance with real picture of bending device; (**g**) cycling stability of the SSHSc device; (**h**) Nyquist plots of before and after cycling stability of the pouch types SSHSc device; (**i**) Ragone plots of the pouch types SSHSc device. Reproduced from Ref. [99]. Copyright 2021, Elsevier Ltd.

The authors of the study [99] also used NiCo_2_O_4_ oxide with spinel structure to fabricate supercapacitor electrodes. Synthesis of the indicated substance was carried out as follows: to the solution of nickel and cobalt nitrates, a solution based on cobalt chloride and urea was added, after which the reaction system was kept at different temperatures (298, 323, 348, and 373 K) for 12 h. The resulting solid phase was separated from the mother liquor by vacuum filtration and further subjected to drying in a vacuum oven at 80 °C for 12 h. The formation of thin films based on thus obtained NiCo_2_O_4_ oxide on the Ni foam surface was carried out using screen printing, followed by drying at room temperature and further heat treatment at 100 °C for 6 h. In addition, a composite based on NiCo_2_O_4_ and N-doped carbon nanotubes was fabricated to improve the functional properties of the formed electrodes. The obtained material showed good electrochemical characteristics (303 mA·h/g at 5 mVs^−1^). Thus, for additional improvement of operational properties of flexible micro-supercapacitor electrodes, composites with the addition of carbon nanostructures, conducting polymers, and other components are formed on the basis of individual metal oxides or oxides of more complex composition (as a rule, with spinel structure) [100,101,102]. As a result, by combining oxide nanostructures with other types of materials as well as using printed technologies, more efficient miniature devices characterized by competitive electrochemical performance can be fabricated (Figure 3).

### 2.3. Polymers

Polymers are a particularly attractive class of materials for advanced supercapacitors, especially flexible and wearable supercapacitors, where there are special requirements for mechanical material properties while maintaining basic device functionality. The advantages of polymers include properties such as viscoelasticity and high resilience to various types of deformation. In addition, the possibility of modification of polymer chains, for example, by grafting various functional groups, and also the combination of polymeric material with materials of other types allows us to obtain new polymeric materials and composites on their basis, characterized by the required level of such important properties for modern supercapacitors as self-healability, electrical conductivity, electrochemical activity, and mechanical strength [103]. Due to these features, different types of polymers are now actively used in the creation of supercapacitors as substrates, various binding additives in the manufacture of functional inks, electrolytes, sealing coatings, and the active material of electrodes [7,63,104].

Polymers used as substrates (e.g., polyethylene terephthalate (PET), polyethylene naphthalate (PEN), polyimide (PI), polydimethylsiloxane (PDMS), etc.) are characterized by chemical resistance, bending strength (up to 180° while maintaining high capacity), water resistance, and different degrees of transparency, which is often important for the development of microelectronics devices [32]. However, it should be taken into account that the above materials are non-conductive, which requires the application of conductive layers of current collectors; in addition, some polymeric substrates (e.g., PDMS) may have problems with wettability by aqueous inks, which leads to inhomogeneity of the coatings formed on their surface as well as their poor adhesion [7]. In addition, polymeric substrates as part of wearable devices are not always convenient when placed on the body, nor are they always biodegradable, which may place additional demands on their recycling.

The use of polymer binders (polyvinylidene fluoride (PVDF), cellulose, chitosan, lignin, poly-3,4-ethylenedioxythiophene (PEDOT), polyaniline (PANI), etc.) in the formation of micro-supercapacitors can help to significantly adjust such parameters of the formed functional coatings as adhesion to the substrate; improved contact between solid phase particles, which contributes to increased electrical conductivity of the final planar structures, homogeneity, and (often) flexibility and mechanical strength of formed films; and increased capacitive and cyclic characteristics of electrode structures [105,106,107,108]. Moreover, the use of such binders allows us to change the viscosity of the resulting inks within considerable limits, adjusting them to the peculiarities of a particular printing method. However, it should also be remembered that the concentration and type of polymer binder in the ink composition should be clearly controlled, as their presence, especially in high concentrations, can negatively affect the resistivity values of the printed target coatings as well as influence their microstructure [109].

Polymer-based electrolytes can be divided into three groups: (a) solid-state electrolytes (e.g., based on Nafion [110], polyaryletherketone [111]); (b) polymer gel electrolytes, which are further divided into hydrogel electrolytes (water as the solvent), e.g., systems based on polyvinyl alcohol (PVA), polyacrylamide (PAM), biopolymers (chitosan, agarose) [109], and organogel electrolytes (organic solvent is used, e.g., systems based on poly(acrylic acid-co-vinylimidazole) in ethylene glycol [112] and poly(vinylidene fluoride-co-hexafluoropropylene) in acetone [113]); and (c) polymer-ionic liquid electrolyte, which are melts (melting point below 100 °C) of organic salts consisting of highly asymmetric anions and cations distributed in a polymer matrix [114,115]. When developing polymer-based electrolytes, it is necessary to consider not only the value of their ionic conductivity, but also the degree of ion selectivity. In addition, the stability of such electrolytes can be significantly reduced when concentrated acids and alkalis are introduced into the polymer matrix. In addition, ensuring the long-term operation of polymer-based electrolytes requires the packaging with sealing layers, which must also possess mechanical strength while maintaining the required degree of flexibility.

Polymers used as active electrode material can be categorized into conducting polymers (polypyrrole (PPy), PANI, polythiophene and its derivatives, redox conducting polymers) [103,116], composites based on conducting polymers (polymer–polymer: PEDOT:PSS [117], PEDOT:PSS/poly(ethylene glycol) diacrylate (PEGDA) [118], etc.; polymer–carbon material: PANI/C-MWCNTs [119], PANI/fullerene [120], PEDOT:PSS/CNT [121], etc.; polymer–metal oxide: PEDOT:TREN:MnO_2_@MnCO_3_ [122], Zn/PPy [123], etc.), and non-conductive polymers (elastomers: PDMS, polyurethane (PU), etc. [124,125]; and non-elastomers: materials based on natural polymers, such as cellulose [5,126]). It can be noted that electrodes based on conducting polymers and their composites demonstrate a pseudo-capacitive mechanism of charge accumulation. Currently, active research is underway to develop technological approaches to create miniaturized flexible/carrier supercapacitors based on polymer materials using inkjet printing, screen printing, and 3D printing. Thus, in the work by Pan et al. [116], an approach to the formation of a micro-supercapacitor based on PANI electrodes formed on the surface of carbon cloth using inkjet printing was considered. The electrode structures were printed using two types of solutions applied to the substrate sequentially: ammonium persulfate in deionized water (solution A) and a mixture of phytic acid (as a gelling agent), aniline, and deionized water (solution B). Next, the electrode structures were held in air for 6 h to form a hierarchically organized hydrogel film and then washed in deionized water for 24 h. The coatings obtained by this method were characterized by high electrical conductivity (about 0.11 S/cm) and specific gravimetric capacitance (∽480 F/g) as well as high charge-discharge rate along with significant cyclic stability (retention of 83% of capacitance after 10,000 operation cycles was observed).

Xu et al. [127] conducted a study devoted to inkjet printing of composite electrode structures based on graphene platelets and PANI on carbon fabric. The approach to the preparation of functional printing ink was as follows: graphene powder and sodium dodecylbenzene sulfonate surfactant were dispersed in water by ultrasonic treatment, and then, PANI was added to the resulting suspension and further treatment by ultrasonic treatment ensued. The unstable fraction was separated using centrifugation and the remaining supernatant was further used as printing ink. The final supercapacitor formed from the above electrode structures was characterized by a specific gravimetric capacitance of 82 F/g, a power density of 124 kW/kg, and an energy density of 2.4 W∙h/kg at a scan rate of 20 mV/s.

**Figure 4:**
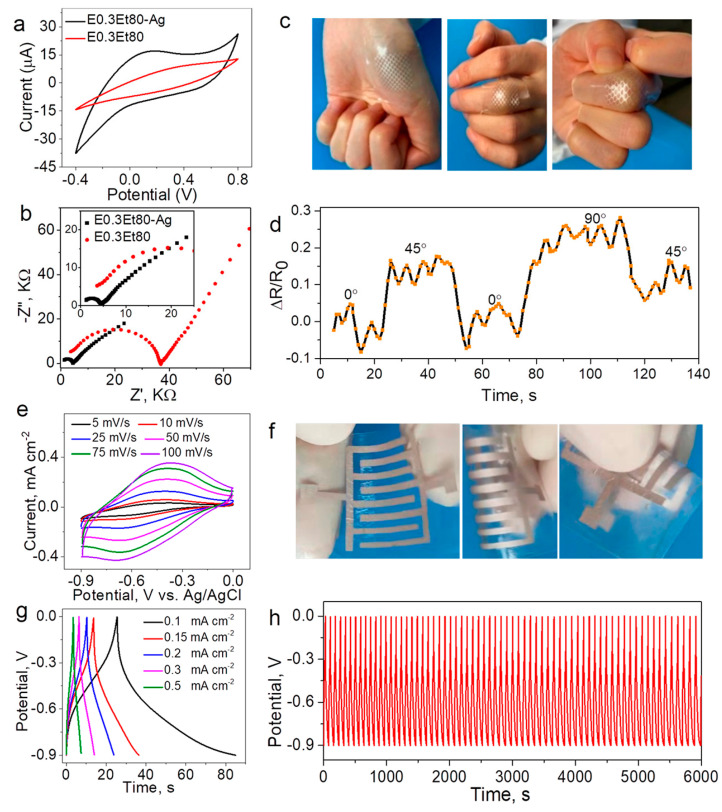
(**a**) Cyclic voltammetry (CV) curves and (**b**) the Nyquist plot for the electrochemical characterization using E0.3Et80 hydrogel and screen printed E0.3Et80 hydrogel-silver (E0.3Et80-Ag) as electrodes in deionized water. (**c**) Digital photographic image showing dual cross-linked chitin hydrogel-printed electronics attached to the skin surfaces and (**d**) changes in the resistance curves on bending the finger at different angles. (**e**) CV curves of the silver supercapacitor at different scan rates in a 1 M Na_2_SO_4_ electrolyte solution. (**f**) Digital photograph showing a foldable and flexible silver SC using the screen-printed E0.3Et80 chitin hydrogel. (**g**) Galvanostatic charge−discharge (GCD) curves at different current densities and (**h**) GCD-curve cycles at a current density of 0.1 mA cm^−2^. Reproduced from Ref. [128]. Copyright 2023, American Chemical Society.

Wang et al. [128] developed a method of sequential chemical and physical crosslinking to synthesize dual cross-linked chitin hydrogels with high strength, flexibility, and biodegradability. It was shown that the obtained hydrogels can be used directly as an electrode material and also as promising flexible substrates for screen printing of electrode structures of miniature supercapacitors on their surface. In particular, it was demonstrated that the formed polymer sample with printed silver electrodes exhibits areal capacitance of 40.8 mF/cm^2^ at a current density of 0.1 mA/cm^2^ as well as high stability after 6000 charge–discharge cycles (Figure 4).

Selvam [122] reported the fabric-based wearable miniaturized supercapacitor fabrication by screen printing a complex composite electrode with the composition of poly3,4-ethylenedioxythiophene: tris(2-aminoethyl)amine: MnO_2_@ MnCO_3_ (PEDOT:TREN:MnO_2_@MnCO_3_). The use of human sweat as an electrolyte was considered. It was shown that the synergetic effect of materials of different types in the composition of electrodes allowed it to achieve not only impressive areal capacitance (30 F/cm^2^) of planar micro-supercapacitor on their basis, but also impressive indicators of cyclic stability (preservation of 92% of capacitance after 50,000 working cycles).

**Figure 5:**
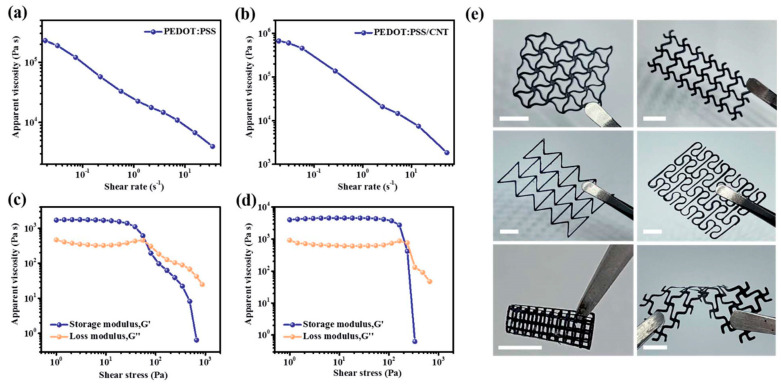
The rheological behaviors of printable inks and digital images of 3D-printed conducting polymer electrodes. Apparent viscosity as a function of shear rate for (**a**) PEDOT:PSS and (**b**) PEDOT:PSS/CNT inks. The storage modulus, G′, and loss modulus, G″, as a function of shear stress for (**c**) PEDOT:PSS and (**d**) PEDOT:PSS/CNT inks. (**e**) Images of as-printed electrodes with different structures. Scale bars: 5 mm. Reproduced from Ref. [121]. Copyright 2021, The Royal Society of Chemistry.

In [118,121], the efficiency of using different types of 3D printing in creation of micro-supercapacitors based on polymer electrodes was demonstrated. In particular, Yang et al. [121] developed additive-free free-standing stretchable electrodes based on PEDOT:PSS/CNT (Figure 5). The functionalized inks were prepared according to the following procedure: in the first step, a commercial aqueous solution of PEDOT:PSS was held in liquid nitrogen for 36 h to form PEDOT:PSS nanofibrils, which were then dispersed in deionized water (DI) or a solvent mixture (DMSO:DI = 15:85 *v*/*v*) followed by stirring and ultrasonic treatment. Next, a commercial CNT paste was added to the resulting dispersion to obtain the final functional ink for electrodes 3D-printing. The internal diameter of the nozzle used for coating was 100 μm, and the printing speed was 2 mm/s.

The electrode structures were printed on the surface of glass substrates, after which the formed structures were immersed in liquid nitrogen with subsequent freeze drying in a vacuum chamber for 10 h to separate them from the substrate. Next, PVA-H_2_SO_4_ gel-polymer electrolyte was applied to the surface of the free-standing electrodes for the final assembly of the micro-supercapacitor. It was shown that the obtained devices exhibit high specific capacitance (990 mF/cm^2^), flexibility (achieved bending up to 180°) and stretchability (maximum elongation by 150%), and good cyclic stability with retention of 74.7% capacitance after 14,000 charge–discharge cycles.

Bertana et al. [118] combined conductive PEDOT with poly(ethylene glycol) diacrylate (PEGDA) in the formation of electrode structures to obtain a composition suitable for stereolithographic printing of the corresponding structures on the surface of platinum current collectors on an aluminum substrate. The PEGDA:PEDOT electrodes printed in this way possessed an electrical conductivity on the order of 200 mS/cm and an energy density of 0.68 μW∙h/cm^2^.

Thus, it can be said that, although polymeric materials show higher values of specific capacitance compared to electrodes based on carbon materials, the problem of cyclic stability as well as the kinetics of charge–discharge processes of polymeric electrodes still requires attention. Possible ways of its solution lie in the field of design of the polymer pore structure as well as the search and development of new composite materials based on them, for example when combining polymers with carbon nanotubes or transition metal oxides.

### 2.4. Metal Hydroxides

**Figure 6:**
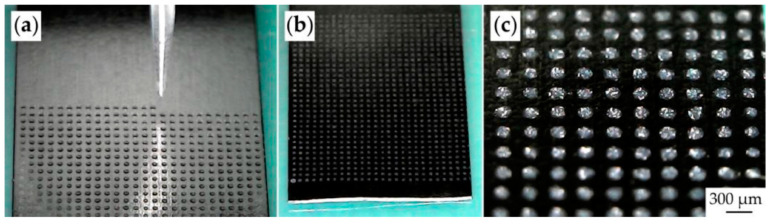
The microplotter printing of functional film (**a**) and the appearance of the resulting miniature planar nanostructure array on a Ni substrate (**b**,**c**). Reproduced from Ref. [138]. Copyright 2023, MDPI.

**Figure 7:**
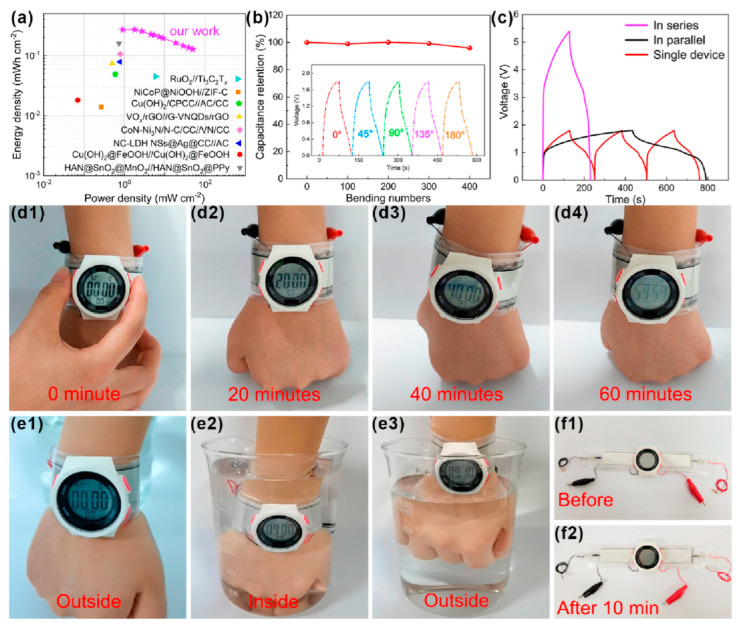
(**a**) Ragone plot compared with the one previously reported. (**b**) Capacitance retention of the flexible Ni_3_Co_1_ LDH@G//AC ASC under different bending cycles (inset: GCD curves tested under different bending angles). (**c**) GCD curves of Ni_3_Co_1_ LDH@G//AC ASC in series and in parallel. (**d1**–**d4**) Demonstration of two flexible Ni_3_Co_1_ LDH@G//AC ASCs connected in series to power the electronic watch. (**e1**–**e3**) Two series-connected flexible Ni_3_Co_1_ LDH@G//AC ASCs are completely immersed in water to power the electronic watch. Demonstration of the device before (**f1**) and after (**f2**) 10 min of immersion in water. Reproduced from Ref. [141]. Copyright 2022, American Chemical Society.

Transition metal hydroxides are also a fairly widespread class of materials for supercapacitor electrodes. Due to its good pseudocapacitive performance and environmentally benign nature, one of the most common in this context is nickel hydroxide [129,130,131]. Thus, Ni(OH)_2_ has a high value of theoretical specific capacitance (2082 F/g in a potential window of 0.5 V). However, the rather low electrical conductivity of this material and small specific surface area usually do not allow to achieve high real pseudocapacitive characteristics. For the formation of flexible supercapacitors containing nanoscale Ni(OH)_2_ structures as an electrode component, printing technologies are also applied, which contributes to the homogeneity of the material and reproducibility of the characteristics of the obtained devices. For example, the authors of the study [132] studied the formation of electrodes based on Ni(OH)_2_ nanoflakes using inkjet printing. The obtained asymmetric supercapacitor exhibited high energy density and power density per unit power (64.8 W·h/kg at 800 W/kg and 30.7 W·h/kg at 16,000 W/kg). In the fabrication of flexible electrodes by screen printing, Ni(OH)_2_ nanoplates were used in [133] while the authors of [134] formed a nickel hydroxide shell on the surface of carbon fibers for further fabrication of micro-supercapacitor electrodes by 3D printing. As can be seen, many researchers are striving to achieve a more developed surface of Ni(OH)_2_ nanostructures, which contributes to the improvement of their electrochemical characteristics and partially compensates for the relatively low electrical conductivity. Much rarer are works that utilize cobalt hydroxide as a component of supercapacitor electrodes [135]. In particular, the authors of [136] studied the fabrication process of β-Co(OH)_2_-based thin-film supercapacitor electrodes using screen printing. The synthesis of cobalt (II) hydroxide in this case was carried out by the hydrothermal method: monoethanolamine was added to an aqueous solution of cobalt (II) chloride hexahydrate (CoCl_2_·6H_2_O), hexamethylenetetramine (HMT), and cetyltrimethylammonium bromide (CTAB), after which the reaction system was transferred to a steel autoclave with Teflon insert and subjected to heat treatment at 100 °C for 24 h. The formed solid phase was further dried at 80 °C in air. Based on the β-Co(OH)_2_ powder thus obtained, an asymmetric supercapacitor was fabricated by screen printing, showing a specific capacitance of about 170 F/g at a current density of 0.5 mA. An aqueous KOH solution (3 mol/L) was used as the electrolyte in this case, and after 600 charge-discharge cycles, a capacity retention of 99.69% was observed. Recently, in order to improve the functional characteristics of hydroxide-based supercapacitor electrodes, a tendency to use materials of more complex composition has arisen, including those characterized by a layered structure. Thus, much attention in this context is paid today to the layered hydroxides (LH), which have a unique structure where charge-balancing anions are located in the interlayer space of metal hydroxides [137]. Layered double hydroxides (LDH), which have much higher electrical conductivity compared to individual hydroxides, attract the most attention. A very popular representative of LDHs is nickel-cobalt hydroxide (NiCo-LDH), which is actively used in the fabrication of electrodes of miniature supercapacitors, including printed technologies [138] (Figure 6). In [139], the formation of an electrode based on the above material on the surface of nickel foam was carried out by the authors using inkjet printing. As a result of electrochemical measurements, it was found that the obtained material combined with reduced graphene oxide exhibits a capacity of about 227 mA·h/g at 1.2 A/g. Inkjet printing is also used in modifying the substrate (including carbon paper) with reduced graphene oxide and silver nanoparticles and then forming NiCo-LDH nanosheets on its surface by electrochemical deposition. The material thus obtained [140] was used as a positive electrode of a supercapacitor, which demonstrated a capacitance of 95 mA h/g at 0.6 A/g and maximum energy density of 76 W·h/kg at a power density of 480 W/kg. When creating electrode structures, great attention is also paid to fabricating a highly developed surface for the prepared materials, which allows for a significant improvement in the electrochemical properties of the device by facilitating the access of the electrolyte and accelerating the diffusion of ions into the structure of the active material. For example, the authors of the study [141] showed the effectiveness of using hierarchical porous layered nickel-cobalt hydroxide in the fabrication of high-performance flexible supercapacitors (Figure 7). In this case, the authors varied the ratio of Ni and Co in the preparation of metal hydroxide, and as a result, they formed nanoflower-like structures. The synthesis was carried out by the hydrothermal method: in a typical experiment, 0.218 g of Co(NO_3_)_2_·6H_2_O, 0.654 g of Ni(NO_3_)_2_·6H_2_O, and 1.0 g of cetyltrimethylammonium bromide were dissolved in a mixture of deionized water (12 mL) and ethyl alcohol (60 mL), after which the reaction system was subjected to heat treatment in a steel autoclave with Teflon liner at 180 °C for 2 h. The resulting solid phase was separated from the mother liquor, washed, and dried at 60 °C. The electrode structures were formed by screen printing, and the resulting electrodes demonstrated specific capacitance of 1575 F/g at 1 A/g as well as 86.3% capacity retention from 1 to 50 A/g.

A number of researchers are also studying other layered double hydroxides, which, like the most common NiCo-LDH, show good characteristics as components of supercapacitor electrodes. Particularly, the authors of [142] use Co-Al layered double hydroxide in the fabrication of micro-supercapacitors by screen-printing method, which in combination with MXene of Ti_3_C_2_T_x_ composition (as a negative electrode) allows for achieving high energy density (8.84 μW·h/cm^2^). In addition, the material has high flexibility and cycling stability (initial capacity is maintained at 92% after 10,000 cycles). There is also research [143] related to the study of a hierarchically organized Ni-V layered double hydroxide (nickel-vanadium hydrotalcite) supercapacitor electrode, which exhibited a specific capacitance of 1069 F/g at 1 A/g, and after 1500 charge-discharge cycles at 20 A/g, this parameter remained at 68%. In addition to the materials discussed above, amorphous FeOOH [76], which in combination with MnO_2_ is characterized by high specific capacitance and good rate capability (350.2 F/g at 0.5 A/g and 159.5 F/g at 20 A/g), was also used for printing supercapacitor electrodes. It was shown that after 10,000 charge-discharge cycles, the specific capacitance is retained at 95.6%, and the all-printed solid-state flexible supercapacitor has a specific capacitance of 5.7 mF/cm^2^ while retaining 80% of this value after 2000 charge-discharge cycles.

Thus, it should be noted that metal hydroxides are in great demand and are promising materials for use as components of flexible micro-supercapacitors, and the efficiency of these devices depends to a large extent on the chemical composition, crystal structure, and microstructural features of the corresponding electrode materials.

### 2.5. Sulfides

Recently, in the search for new more efficient supercapacitor electrodes, many researchers have increasingly turned their attention to metal sulfides, which often show improved functional characteristics compared to the corresponding oxides. For example, when replacing oxygen in such a highly popular and demanded oxide as NiCo_2_O_4_ with sulfur, the resulting sulfide of NiCo_2_S_4_ composition demonstrates even more outstanding properties [144]. In particular, in this case, there is an increase in the length of chemical bonds leading to easier electron transport, which may contribute to improved electrochemical performance. As a result, two orders of magnitude increase in electrical conductivity is observed for NiCo_2_S_4_ sulfide compared to NiCo_2_O_4_ oxide, and higher electrochemical activity and specific capacitance are observed. Due to the mentioned features of nickel–cobalt sulfide, this material is one of the most demanded sulfides as a component of supercapacitor electrodes [145,146]. In addition, researchers are also interested in a sulfide with the opposite ratio of metals of the composition CoNi_2_S_4_, which also shows high electrochemical properties as an electrode of supercapacitors [147,148]. One of the most popular pseudocapacitance sulfide materials in this context is also MoS_2_, which is characterized by a graphene-like two-dimensional layered structure and sandwich-structured S-Mo-S atoms held together by weak van der Waals forces [149]. Due to the peculiarities of the structure, materials based on molybdenum disulfide can reach high values of a specific surface area, which promotes charge storage of EDLCs and also provides the possibility of Faraday redox reactions on molybdenum atoms with different oxidation degree (from +2 to +6). Thus, semiconducting molybdenum disulfide can be considered as one of the most promising materials for supercapacitor electrodes, which has a high theoretical specific capacitance (1000 F/g).

**Figure 8:**
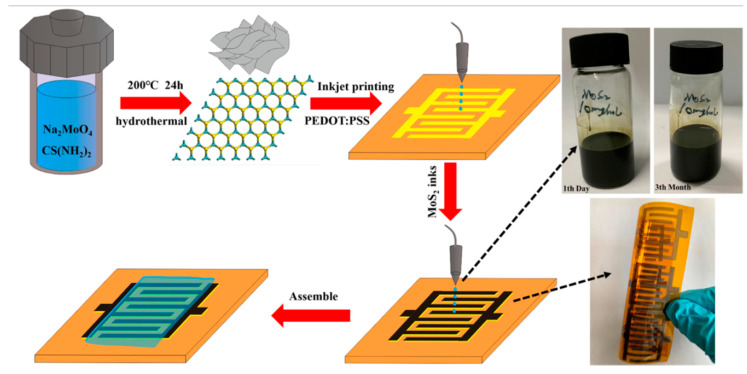
Schematic fabrication of flexible MSCs using MoS_2_ nanosheets; the inset shows the stability and optimization of MoS_2_ inks for 1 day and after 3 months and the digital photograph of flexible 10 L interdigital electrodes printed with a desktop inkjet printer on the PI substrate. Reproduced from Ref. [149]. Copyright 2020, American Chemical Society.

Moreover, the layered structure of MoS_2_ allows for the formation of thinner and more flexible micro-supercapacitors compared to traditional vertical MSCs, including those using printed technologies. For example, the authors of [149] convincingly demonstrated the efficiency of inkjet printing in the fabrication of a flexible supercapacitor using MoS_2_ nanosheets (Figure 8). Synthesis of molybdenum disulfide in this case was carried out by hydrothermal method: the reaction system, which is a solution of sodium molybdate (Na_2_MoO_4_, 484 mg) and thiourea (CH_4_N_2_S, 304 mg) in deionized water (40 mL), was transferred to an autoclave, which was further placed in a muffle furnace at 200 °C for 24 h. To prepare the ink, the obtained molybdenum disulfide nanosheets were dispersed in 10 mL of a mixture of isopropyl alcohol and water (volume ratio 4:1) in the presence of a non-ionogenic surfactant (polyvinylpyrrolidone), which reduces the probability of the obtained MoS_2_ nanosheets agglomeration during the formation of electrodes. In addition, 1 mL of propylene glycol was added to the substrate to optimize the properties of the ink and slow its drying time. The MoS_2_ interdigital electrodes were printed onto the surface of a polyimide substrate using a consumer desktop inkjet printer. In the manufacturing of the device, a gel electrolyte was used, which was a solution of 4 g H_2_SO_4_ (98%) and 4 g polyvinyl alcohol in 40 mL of deionized water. As a result of electrochemical measurements, it was determined that the obtained flexible micro-supercapacitors have high surface capacitance (175 μF/cm^2^ at 4 μA/cm^2^), corresponding to a maximum energy and power density of 0.215 mW h/cm^3^ and 0.022 W/cm^3^, respectively. It was also shown that the obtained device has high cyclic stability: after 10,000 cycles, the specific capacitance remained at 85.6%. The authors of the study [150], in order to suppress the agglomeration process of molybdenum disulfide particles in hydrothermal conditions and added PEDOT: PSS and due to the electrostatic interactions between the negatively charged MoS_2_ particles and the positively charged PEDOT chain, the formation of a hybrid hydrogel occurred, which could be used as a functional ink in extrusion 3D printing of flexible supercapacitors, including miniature ones. The materials thus obtained showed good mechanical properties (fracture strength of 18.59 MPa) as well as electrochemical performance in aqueous Na_2_SO_4_ electrolyte (474 mF/cm^2^) and solid-state PVA−H_3_PO_4_ electrolyte (360 mF/cm^2^). Other printing technologies, such as spray printing [151], gravure printing [152], and screen printing [153], are also used to fabricate MSCs electrodes based on molybdenum disulfide. MoS_x_ and MoS_3−x_ based electrode structures were also fabricated using 3D printing [154,155] and showed good electrochemical performance, which further indicates the significant prospects of molybdenum sulfides in the development of micro-supercapacitors.

It should be noted that there are also works devoted to studying the properties of CuCo_2_S_4_ sulfide as a new electrode material for supercapacitors. Thus, replacement of nickel in the previously mentioned NiCo_2_S_4_ sulfide with more common copper can contribute to the reduction of material cost while maintaining competitive electrochemical characteristics [156]. Authors of [157] used this sulfide as a component of supercapacitor electrodes in their fabrication by 3D printing. The synthesis of the material in this case was carried out by hydrothermal process in the presence of reduced graphene oxide using copper and cobalt chlorides and thiourea. Heat treatment of the reaction system was carried out in an autoclave at 200 °C for 12 h. 3D printing of the supercapacitor electrode in this case was carried out with gel freezing, which, according to the authors, contributed not only to the formation of porous material, but also to the suppression of agglomeration of solid phase particles to improve charge transport. Electrochemical measurements allowed for establishing that the printed electrode has a high specific capacity (C_sp_ = 1123 F/g), and after 20,000 charge-discharge cycles at high current density (125 A/g), the value of this parameter remained at the level of 91.2%. It was also shown that the investigated electrode has low internal resistance, low ion exchange resistance, and high electric double layer capacitance.

Significant attention in the manufacture of supercapacitor electrodes is also paid to the sulfide MnCo_2_S_4_. In particular, the authors of [158] studied the process of forming the corresponding electrodes using 3D printing. The synthesis of manganese–cobalt sulfide was carried out by a thermal decomposition method with 1-dodecanethiol, which plays the role of both sulfur source and surfactant. For this purpose, manganese and cobalt chlorides in the required ratios were placed in a three-neck flask containing 1-octadecene and vacuumized at room temperature for 30 min. Then, the reaction system was heated to 140 °C, dodecanethiol was quickly introduced, and the temperature was raised to 290 °C and kept for 1 h, with system being stirred. According to the electrochemical measurements of the obtained electrode, the specific capacitance values were 3812.5 F/g (at 2 A/g) and 1780.8 F/g (at 50 A/g). The results of the electrode cyclic stability study showed a capacity retention of 92% after 22,000 charge–discharge cycles (at 50 A/g). In [159], the authors studied the fabrication process of MnCo_2_S_4_-based electrodes using screen printing. Synthesis of sulfide in this case was carried out by chemical precipitation by interaction of manganese and cobalt sulfates with sodium sulfide followed by heat treatment of the material at 300 °C in argon atmosphere.

The authors of the study [160] used KCu_7_S_4_ nanorods as a component of micro-supercapacitor electrodes, and the material was fabricated using 3D-printing. The obtained electrode exhibited a gravimetric capacitance of 815.83 F/g at 0.5 A/g, corresponding to surface and bulk capacitance of 7.33 F/cm^2^ and 27.7 F/cm^3^.

Thus, quite a wide range of metal sulfides show themselves as promising components of MSCs electrodes, in connection with which they have recently attracted increasing interest of the scientific community.

### 2.6. MXenes

It is well known that MXenes are a rather new and extensive class of 2D nanomaterials with the general formula M_n+1_X_n_T_x_, where M is the transition metal (most commonly, Ti, V, Nb, Mo, Cr, etc.), X is C or N, and T is the surface functional groups, most commonly F, OH, Cl [161,162]. Due to their layered structure, high electrical conductivity, and huge variation in composition (which allows its optimization for a specific task), MXenes are recognized as very promising component base for various modern devices, e.g., in sensorics [163,164,165,166,167], catalysis [168,169], industrial water purification [170,171], and the creation of electrodes that often retain high energy density at high current densities [172]. However, they are of the greatest interest as components of energy generation and storage devices—lithium/sodium-ion batteries and supercapacitors [173,174,175,176,177]—especially owing to the possibility of intercalation of various ions into the interlayer space and high hydrophilicity of surface groups. An attractive aspect for the development of portable and implantable devices (similar to e-textile or electronic-tattoo) based on MXene is their antibacterial activity and mechanical elasticity of the layers, as established in several studies [178,179]. The development of printing technologies has most significantly affected the issue of MXene-based supercapacitor and micro-supercapacitor electrodes [32,180,181,182].

Many scientific publications indicate that the history of MXene preparation (etching technique of initial MAX phases, reagents used, temperature, duration, delamination conditions) is crucial for the observed electrochemical characteristics as it significantly affects the composition of surface functional groups, the size and shape of formed 2D-particles, the interaction between individual MXene flakes, the interlayer distance, and the possibility of ionic and molecular intercalation. The number of layers in the MXene aggregates formed after etching and delamination can affect not only the electrochemical behavior of the applied coatings, but also the viscosity of the initial ink [183] (which is due to the significantly higher ζ-potential and strong long-range interactions for monolayer MXene compared to multilayer ones). As a result, this parameter can also determine the additive technology used to form the electrode layers: 2D printing (inkjet, extrusion, etc.) or 3D printing/screen printing.

Analyzing the features of MXene-based functional inks, it should be noted that usually, in addition to the electrochemically active component (in this case, MXene), they contain other components that provide the necessary viscosity, binding of particles to each other in the final coating, or surface-active components (to improve wetting of the so-called “pigment” by the dispersion medium). These additives significantly worsen the electrophysical properties, reduce the active surface area and complicate the fabrication process, and their removal most often requires high-temperature treatment (preferably pulsed and localized to prevent degradation of the main components). Due to the high chemical activity of MXenes, great attention is paid to minimizing the content of such additives. In general, the simultaneous high electrical conductivity of MXenes and high concentration of polar surface functional groups in them (as a result of which at pH > 2–3 ζ-potential in aqueous solution is in the range from −30 to −80 mV [184]), providing good hydrophilicity and dispersibility in polar solvents, contribute to the recognition of MXenes as a very promising object for the formation of liquid inks already at room temperature. There is information about the possibility of obtaining stable suspensions of MXenes with solid phase content from 0.02 to 70 wt.%. The presence of hydrogen bonds and van der Waals interactions between individual plates in MXene coatings also allows for minimizing the content of binding components.

The rheological properties of MXene dispersions were found to be strongly dependent on the concentration of the solid phase. Aghayar et al., in their review [180], identified three concentration intervals that determine the features of the rheological behavior of dispersion systems containing MXenes:

1. For ~5 µm MXene multilayer dispersions at low concentrations (10 wt.%), little interaction of randomly distributed particles is observed;
2. For suspensions with medium MXene content (~30 wt.%), a sol-gel transition (viscous liquid at low frequencies and gel at high frequencies) can be observed;
3. At high MXene contents (>40 wt.%), a colloidal gel with percolated network of nanosheets is formed, which is characterized by a significant flow stress [183].

At the same time, low-concentration MXene inks, mainly used in inkjet printing, are most often used for the application of transparent electrodes with high charge storage capacity. At the same time, it is noted [185] that for energy storage devices, it is considered more convenient to use more concentrated inks with the use of the extrusion printing method. For example, this paper demonstrates the advantage of three-pass extrusion printing over 25-pass inkjet printing.

While there are positive aspects of using MXene aqueous dispersions as functional inks for the formation of planar and volume components of supercapacitors, there are also objective and significant problems:

1. Oxidative degradation of MXene in aqueous medium during storage and as part of electrochemical devices when immersed in electrolytes containing water;
2. Tendency of MXene delaminated sheets to reaggregate into multilayer aggregates during drying of coatings;
3. Significant differences in MXene properties depending on the synthesis, delamination, and isolation techniques, making it difficult to implement the technologies in production.

Several different approaches have been proposed as a possible solution to the low oxidative stability of MXene-based inks. In particular, making the dispersion using deoxygenated water followed by storage in sealed argon-filled vessels at low temperatures (~5 °C) results in longer ink life. Furthermore, it was found [186] that suspensions and pastes with higher concentration of MXene exhibit better resistance to oxidative degradation compared to diluted ones.

Switching to non-aqueous solvents (most often polar solvents such as DMSO, N-methyl-2-pyrrolidone, ethanol, etc.) also allows us to increase the utilization time of dispersions compared to aqueous solvents. Notably, for dispersions in the above solvents, it was found in a study [187] that their viscosity is generally lower than when water is used as the dispersion medium; however, it may also be due to the higher concentration of MXene that is achievable in the aqueous medium. For example, in the work of Deng and co-workers [186], to form a highly concentrated paste of MXene, Ti_3_C_2_T_x_, not the classical technique of concentrating a dilute dispersion, was used, but a dosed injection into lyophilized after rapid freezing Ti_3_C_2_T_x_ powder via exposure to water mist was performed. It showed high electrical conductivity and retention of properties for two months without noticeable MXene decay (in case of storage at reduced temperature with suppressed hydration). The PVS-H_2_SO_4_ polymer electrolyte coated microcapacitor obtained using this paste exhibited a gravimetric capacitance of 161.7 F/g at 0.05 A/g.

Another efficient way to stabilize the oxidative stability of MXenes in aqueous suspensions is the introduction of organic polydentate ligands [188,189], particularly sodium ascorbate. In these cases, there is not only an improvement in MXene dispersibility, but also an increase in oxidation resistance, probably due to the interaction of ascorbate ions with available titanium atoms through coordination and hydrogen bonding with surface functional groups. This leads to a significant increase in the interlayer distance in MXene and, consequently, to an increase in the charge/discharge rate and specific capacitance characteristics.

A more complex composition modifying MXene Ti_3_C_2_T_x_ is described in the study by Wang et al. [190]: the used sodium alginate in addition to protecting against oxidation serves to build three-dimensional framework structures (and levels the problem of aggregation of low-layer MXenes) and the introduction of Fe^2+^ salt structures of the pore space. With improved dispersion stability, it is characterized by a viscosity suitable for inkjet printing (1619.9 mPa/s). The micro-supercapacitor printed by this method on paper substrate with electrode spacing of 310 μm has a large number of active sites for ion storage and a network with high conductivity for electron transfer, resulting in good performance (capacitance 123. 8 mF/cm^2^ at 5 mV/s, energy density 8.44 μW·h/cm^2^ with a power density of 33.70 μW/cm^2^) with 91.4% capacitance retention after 10,000 cycles and 90% capacitance retention after 10,000 bending cycles.

Doping MXene with nitrogen and sulfur atoms is also considered promising for improving its environmental stability [191] and also helps to improve its electrochemical properties. Thus, using Ti_3_C_2_T_x_, modified with sulfur and nitrogen, planar micro-supercapacitors with electrode spacing of 300 μm were formed with the direct inkjet printing method created by Sun et al., for which a high bulk capacitance of 710 F/cm^3^ and an energy density of 8.9 mW·h/cm^3^ at a power density of 411 mW/cm^3^ was observed. Long-term cyclic stability was also recorded for it (up to 94.6% after 10,000 cycles).

Interesting from the economic and technological points of view is the approach of the authors of the article [192], who proposed to use not monolayer MXene obtained by delamination in DMSO and ultrasonic action and transferred by centrifugation (3500 rpm, 1 h) to the supernatant, but a precipitate containing more multilayer MXene and residues of the MAX-phase Ti_3_AlC_2_ for the fabrication of functional Ti_3_C_2_T_x_ inks. This concentrated dispersed system with a solid phase content of 72.4 wt.% has the required rheological characteristics (viscosity 468 Pa/s at a shear rate of 10^−1^ s^−1^) and exhibits the properties of a non-Newtonian fluid, which is required for extrusion printing. The successful formation at room temperature of a micro-supercapacitor on various substrates (polymer, paper, glass) was demonstrated, as was the subsequent application of PVA-H_2_SO_4_ electrolyte at a drying temperature of 40 °C. The authors claim ultra-high surface capacitance (up to 2337 mF/cm^2^), long-term cycling stability up to 10,000 cycles (93.1% retention at 10 mA/cm^2^), and excellent flexibility (with deformation from 0 to 180 degrees).

**Figure 9:**
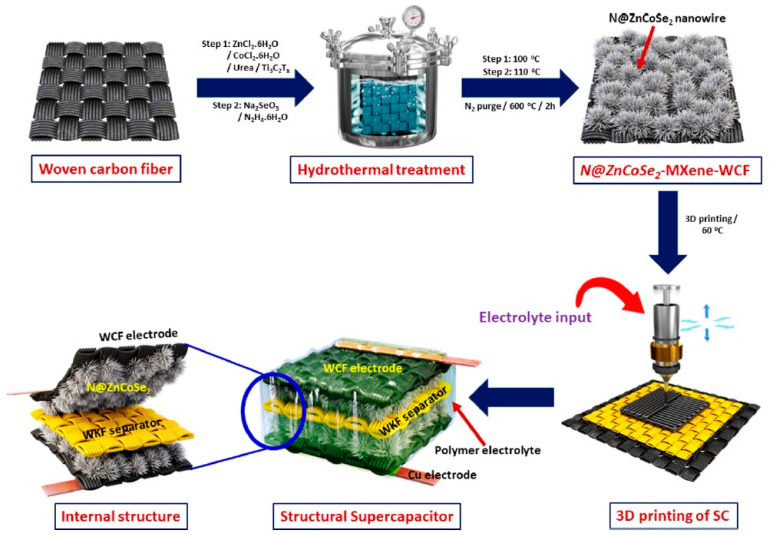
Schematic diagram of MXene-N@ZnCoSe_2_ nanowires on WCF and development of the supercapacitor by the 3D printing process. Reproduced from Ref. [196]. Copyright 2023, American Chemical Society.

Overall, the current vigorous work on finding ways to improve the stability of MXenes in aqueous dispersions opens up prospects for designing more environmentally friendly production of energy storage devices in the future. Furthermore, there is a huge body of research devoted to the study of the effectiveness of improving the characteristics of such devices as a result of the use of composites, for example, with the addition of electrically conductive polymers [193,194], semiconducting oxide, chalcogenide [195,196] and carbon nanoparticles [197], layered double and triple metal hydroxides [198], and other components (Figure 9). This can lead not only to an increase in basic properties (as a result of the formation of three-dimensional structure and heterojunctions), but also due to the synergetic effect to the emergence of new useful qualities, for example, the expansion of the temperature range of application of supercapacitors on their basis both in the direction of high and low temperatures, to an increase in mechanical strength to stretching, impact, etc.

Summarizing, we can say that the use of such a new class of planar nanomaterials as MXenes for the fabrication of supercapacitor components using printed technologies, despite some problems that are actively being addressed, is a very promising and relevant area of research.

### 2.7. Composites

As follows from the previous sections, in most cases, supercapacitor electrodes are formed by combining materials of different types, and the composites created in this way unite the properties of the corresponding components, which makes it possible to achieve optimal mechanical and electrochemical characteristics. Thus, there is a large number of works that consider approaches to obtaining composite electrodes based on all the materials we have noted above (carbon structures, polymers, oxides, hydroxides and metal sulfides, and MXenes). In the fabrication of micro-supercapacitor electrodes using printing technologies, composites based on various polymers and carbon nanostructures, in particular fullerenes, are studied [199]. Thus, with the combination of polyaniline and fullerenes, the composite electrode is characterized by the specific capacitance value of 2201 F/g at a current density of 2 A/g (rate capability of about 73% at 10 A/g) [120]. As noted above, a good result in the context of electrochemical characteristics can be achieved using composites based on MXenes (including Ti_3_C_2_T_x_ composition) and layered double hydroxides (in particular, CoAl-LDH): high energy density (8.84 μW·h/cm^2^), flexibility, and cycling stability (capacitance remains at the level of 92% after 10,000 cycles). As an example of the effective integration of carbon structures with metal oxides, we can cite the study [200], where the authors studied the formation of a composite electrode based on graphene, carbon nanotubes, and MnO_2_.

**Figure 10:**
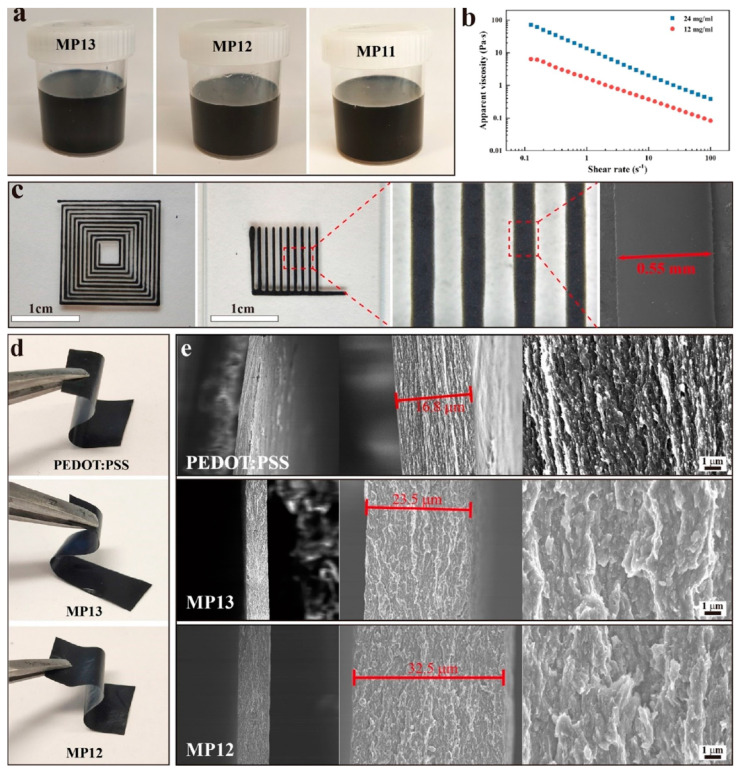
Processability demonstration of MP dispersion. (**a**) Digital image of MP dispersions; (**b**) viscosities of MP12 dispersions (12 mg mL^−1^; 24 mg mL^−1^) as a function of shear rate; (**c**) 3D printed square spiral and comblike patterns using a MP12 dispersion of 24 mg mL^−1^; (**d**) flexibility demonstration; and (**e**) cross-sectional SEM images of the filtered neat PEDOT:PSS, MP13, and MP12 films. Reproduced from Ref. [150]. Copyright 2021, American Chemical Society.

The obtained material demonstrated improved electrochemical characteristics (good mechanical strength, specific capacitance of 103 mF/cm^2^, promising areal energy density of 14.3 μW·h/cm^2^, and capacitance retention at the level of 94% after 8000 charge-discharge cycles). In [150], the results of successful combination of MoS_2_ and PEDOT:PSS in the electrode composition of a flexible supercapacitor are presented (Figure 10). In this case, due to the addition of the above polymer to the material composition, its electrostatic interaction with molybdenum disulfide particles occurred, leading to the formation of a hybrid hydrogel, which can be used as a functional ink in extrusion 3D printing of flexible supercapacitors, including miniature ones. The electrodes thus fabricated showed good mechanical and electrochemical performance in the environment of various electrolytes. As a result, it was found that composite materials are probably the most common and promising as electrodes for micro-supercapacitors formed using classical methods and different types of printing technologies.

Thus, it can be concluded from the performed literature analysis that printing technologies belong to the most promising and dynamically developing technological approaches to fabrication of flexible and miniaturized supercapacitors. Their main advantages compared to classical techniques are the possibility of forming films possessing complex geometry with high reproducibility of microstructural and functional characteristics, accurate and targeted dosage, automation, and a higher speed of desired materials preparation. All the discussed types of electrode materials have their own advantages and drawbacks (Table A1, Appendix A), and so, in the future, the scientific community will have to continue searching for the most universal materials, which would have high electrical conductivity and capacitance parameters, highly developed surface and exhibited high cyclic stability, and being readily available and environmentally friendly. Today, researchers are mostly following the path of creating composite materials that combine advantages of substances of varying nature.

## 3. Conclusions

Supercapacitors, as energy storage devices, will undoubtedly play an important role in the sustainable development of modern energy. Technologies for their creation are being improved for many years already; serious efforts are made to develop functional components of supercapacitors with improved electrochemical characteristics, the search for optimal methods of their automated manufacturing is underway, and more and more effective approaches to miniaturization and planarization of these devices are offered. In the framework of this review, we have considered several of the most popular classes of materials used as active components of micro-supercapacitor electrodes formed using printing technologies. Despite the successes achieved by researchers in this field, there are still a number of challenges that require increased attention.

Thus, in the case of carbon materials, it is necessary to note insufficiently high values of specific capacitance. Although there are attempts to improve this parameter by increasing the specific surface area and adjusting the pore structure, these measures have had limited effect. Significant improvements in the capacitive performance of carbon materials can be achieved when they are combined with pseudo-capacitive materials. However, the power density and cyclic stability of the final materials may suffer in this case. Polymer electrode materials in turn can also suffer from low cyclic stability, mechanical strength, and insufficiently high conductivity. The latter parameter also requires correction for transition metal oxides and hydroxides. When working with metal sulfides, attempts should be made to increase their interlayer space to facilitate charge transfer. In addition, in aqueous electrolyte environments, this type of material cannot function in a wide potential window, whereas degradation is observed in ionic liquids. MXenes, which represent a new class of layered materials, despite their extremely attractive electrochemical properties, require the use of rather complex synthetic approaches, and improvements in oxidative stability during long-term storage as well as operation in aqueous electrolytes are need to be made.

The solution to these problems may lie in the field of composite materials combining different energy storage mechanisms. In addition, the development of efficient and simultaneously uncomplicated synthetic approaches is required to allow the tuning of the necessary microstructural features of the formed materials in order to improve their performance and reduce their cost. Thus, the use of nanomaterials with hierarchically organized microstructure facilitates electrolyte access and accelerates ion diffusion into the structure of the active electrode material. The application of printing technologies allows to advance significantly on the way to automated creation of micro-supercapacitors of different geometric design for the needs of modern microelectronics, but in this context, it is also necessary to solve such problems as the optimization of ink composition (in particular, the presence of binder additives and surfactants that affect the final functional characteristics of the material), level of their viscosity, sedimentation stability, compatibility of inks, and substrates of a different nature.

## Data Availability

Not applicable.

## Abbreviations

AC	activated carbon
ASC	asymmetric supercapacitor
CNO	carbon nano-onions
CNT	carbon nanotubes
CTAB	cetyltrimethylammonium bromide
CV	cyclic voltammetry
DIW	direct ink writing
DMSO	dimethyl sulfoxide
ECSDs	electrochromic supercapacitors displays
EDLC	electric double-layer capacitor
GCD	galvanostatic charge−discharge
HMT	hexamethyl-enetetramine
LDH	layered double hydroxides
LED	light emitting diode
LH	layered hydroxides
MP	MoS_2_/PEDOT:PSS
MSC	micro-supercapacitor
NMP	N-methyl-2-pyrrolidone
OLC	onion-like carbons
OM	optical micrography
PAM	polyacrylamide
PANI	polyaniline
PDMS	polydimethylsiloxane
PEDOT:PSS	poly-3,4-ethylenedioxythiophene:polystyrene sulfonate
PEGDA	poly(ethylene glycol) diacrylate
PEISA	printing-assisted evaporation-induced self-assembly
PEN	polyethylene naphthalate
PET	polyethylene terephthalate
PI	polyimide
PPy	polypyrrole
PSS	polystyrene sulfonate
PU	polyurethane
PVA	polyvinyl alcohol
PVDF	polyvinylidene fluoride
SSHSc	solid-state hybrid supercapacitors
SWCNT	single-walled carbon nanotubes
TREN	tris (2-aminoethyl)amine
WCF	woven carbon fiber
WJM	wet jet milling
WKFs	woven Kevlar fiber

## Appendix A

**Table A1:** Electrochemical characteristics of supercapacitors based on different types of electrode materials.

Electrode Material	Printing Technique	Specific Capacitance	Power Density/Energy Density	Cyclic Stability	Reference
Graphene/SWCNTs	Screen-printing	1.324 mF/cm^2^ at 0.0125 mA/cm^2^	above 20 mW/cm^2^ at 0.064 µW·h/cm^2^	Up to 98% of capacitance was retained after 10,000 cycles at a current density of 0.1875 mA/cm^2^	[61]
SWCNT	Direct Ink Writing	C_vol_ = 15.34 F/cm^3^ at 1.32 A/cm^3^	at 11.8 W/cm^3^ at 1.2 mW·h/cm^3^	97.7% of capacitance after 1000 cycles	[56]
WO_3_	Printing-assisted evaporation-induced self-assembly	C_areal_ = 2.57 mF/cm^2^ at 1.0 mA/cm^2^	0.2 kW/kg at 15.8 W·h/kg	93.8% of capacitance after 1000 cycles	[91]
NiCo_2_O_4_/N-doped CNT@AC	Screen-printing	303 mA·h/g at 5 mV/s	8.96 kW/kg at 12.31 W·h/kg	84% of capacitance after 2000 cycles	[99]
PEDOT:PSS/CNT	3D-printing	990 mF/cm^2^ at 1 mA/cm^2^	0.065 mW h/cm_2_ at 0.4 mW/cm^2^	capacitance retention of 74.7% after 14,000 cycles	[121]
PEGDA:PEDOT	Stereolithography 3D printing process	11.4 mF/cm^2^ at 5 µA/cm^2^	0.002 mW/cm^2^ at 0.68 μWh/cm^2^	capacitance retention of 90% after 500 cycles	[118]
multilayered Ni(OH)_2_/RGO/Ni(OH)_2_/RGO structure	Inkjet printing	1386 F/g at 1 A/g	64.8 W·h/kg at 800 W/kg and 30.7 W·h/kg at 16,000 W/kg	80.4% capacity retention after 5000 cycles	[132]
Ni_3_Co_1_ LDH@graphene	Screen-printing	599 mF/cm^2^ at 1 mA/cm^2^	0.27 mW h/cm^2^ at 49.9 mW/cm^2^	remains 95.8% capacity retention after 400 bending cycles	[141]
MoS_2_ nanosheets	Inkjet printing	175 μF/cm at 4 μA/cm^2^	0.215 mW h/cm^3^ at 0.079 W/cm^3^	85.6% capacitance retention after 10,000 cycles	[149]
MnCo_2_S_4_/rGO	3D-printing	3812.5 F/g at 2 A/g	43.91 W h/kg at 505 W/kg	capacitance retention over 92% after 22,000 cycles	[158]
N, S-doped Ti_3_C_2_T_x_	Inkjet printing	710 F/cm^3^ at 20 μA/cm	8.9 mW·h/cm^3^ at a power density of 411 mW/cm^3^	capacitance retention over 94.6% after 10,000 cycles	[191]
N@ZnCoSe_2_-Ti_3_C_2_T_x_, where Tx = O^−^ or F^−^	3D-printing	19.36 F/g	2.69 W·h/kg at 43.20 W/kg	capacitance retention over 88.8% after 6000 cycles	[196]
